# Calreticulin Mutations in Myeloproliferative Neoplasms: Comparison of Three Diagnostic Methods

**DOI:** 10.1371/journal.pone.0141010

**Published:** 2015-10-26

**Authors:** Ji-Hye Park, Margaux Sevin, Selim Ramla, Aurélie Truffot, Tiffany Verrier, Dominique Bouchot, Martine Courtois, Mathilde Bas, Sonia Benali, François Bailly, Bernardine Favre, Julien Guy, Laurent Martin, Marc Maynadié, Serge Carillo, François Girodon

**Affiliations:** 1 Service d’Hématologie Biologique, Pôle Biologie, CHU de Dijon, Dijon, France; 2 Inserm U866, Université de Bourgogne, Dijon, France; 3 UMR CNRS 5247, Université de Montpellier, Montpellier, France; 4 Laboratoire de Cytologie Clinique, Pôle Biologie, CHU de Nîmes, Nîmes, France; 5 CRB Ferdinand Cabanne, Dijon, France; 6 Service d’anatomo-pathologie, CHU de Dijon, Dijon, France; Queen's University Belfast, UNITED KINGDOM

## Abstract

Calreticulin (*CALR*) mutations have recently been reported in 70–84% of *JAK2V617F*-negative myeloproliferative neoplasms (MPN), and this detection has become necessary to improve the diagnosis of MPN. In a large single-centre cohort of 298 patients suffering from Essential Thrombocythemia (ET), the *JAK2V617F*, *CALR* and *MPL* mutations were noted in 179 (60%), 56 (18.5%) and 13 (4.5%) respectively. For the detection of the *CALR* mutations, three methods were compared in parallel: high-resolution melting-curve analysis (HRM), product-sizing analysis and Sanger sequencing. The sensitivity for the HRM, product-sizing analysis and Sanger sequencing was 96.4%, 98.2% and 89.3% respectively, whereas the specificity was 96.3%, 100% and 100%. In our cohort, the product-sizing analysis was the most sensitive method and was the easiest to interpret, while the HRM was sometimes difficult to interpret. In contrast, when large series of samples were tested, HRM provided results more quickly than did the other methods, which required more time. Finally, the sequencing method, which is the reference method, had the lowest sensitivity but can be used to describe the type of mutation precisely. Altogether, our results suggest that in routine laboratory practice, product-sizing analysis is globally similar to HRM for the detection of *CALR* mutations, and that both may be used as first-line screening tests. If the results are positive, Sanger sequencing can be used to confirm the mutation and to determine its type. Product-sizing analysis provides sensitive and specific results, moreover, with the quantitative measurement of *CALR*, which might be useful to monitor specific treatments.

## Introduction

In 2005, the diagnosis of Myeloproliferative neoplasms (MPN) was improved by the discovery of *JAK2*V617F, *MPL* exon 10, *JAK2* exon 12 mutations [1–5]. However, despite feasible genetic tests used in current practice, nearly 40% of Essential Thrombocythemia (ET) and Primary Myelofibrosis (PMF) had no specific molecular marker. Recently, calreticulin (*CALR*) mutations have been reported to cover 70 to 84% of *JAK2* and *MPL*-negative ET and PMF [6,7], thus completing the molecular signature of MPN.

All *CALR* mutations involve exon 9 and are somatic insertions or deletions causing a frameshift to the alternative reading frame. Two mutation variants (type 1 and type 2 [6]) are the most frequent: type 1 (L367fs*46) results from a 52-base-pair (bp) deletion and type 2 (K385fs*47) results from a 5-bp TTGTC insertion. These mutations result in a significantly altered C-terminal, in which most of the acidic domain and KDEL signal are lost (ER retention motif). Thus, the detection of *CALR* mutations is of interest in the diagnosis of MPN [8]. The aim of our study was to compare in parallel three diagnostic methods, [High Resolution Melting (HRM) analysis, product-sizing analysis and Sanger sequencing] for the detection of *CALR* mutations in a large single-centre cohort of ET patients including 56 *CALR* mutated cases.

Similar studies on this topic have already been published; however, most of these involved small series of patients (n<22 *CALR* mutated cases) and did not include rare *CALR* mutations [9–11]. On the other hand, in the very good paper of Jones and coll [12], the DNA samples tested were initially selected using Sanger sequencing analysis. Given the low sensitivity of Sanger sequencing (10–25%), it is likely that mutations not detected by Sanger were not tested. In contrast, our study included all blind triple negative ET, which allowed us to compare *CALR* mutations not detected by Sanger sequencing. Our study therefore provides original results on these particular mutations (in our study, these mutations represented 3.5% of the all *CALR* mutations).

## Material and Methods

The procedures followed were in accordance with the Declaration of Helsinki, and samples were obtained following patients’ written informed consent. The data bank is registered with the number # 1255461in the CNIL (national data protection agency) and an ethical approval was obtained by the institutional ethics committee “Comité de Protection des personnes EST I”. Prior to analysis, all samples were anonymized and de-identified.

A total of 298 ET patients from the haematology laboratory, diagnosed according to 2008 WHO diagnostic criteria [13] at the University Hospital of Dijon, France, were retrospectively studied.

Purification of granulocytes and peripheral blood mononuclear cells (PBMCs) and extraction of DNA were performed as previously described [14]. The *JAK2*V617F mutation was analysed by allele-specific real-time quantitative PCR to estimate the *JAK2*V617F mutated allele burden according to the method published by Lippert *et al*. [14] with a sensitivity <1%. The *MPL* mutations were analysed by High Resolution Melting (HRM) curve analyses followed by Sanger sequencing if positive, as reported by Boyd *et al*. [15]. The *CALR* exon 9 mutations were screened by either HRM (adapted from Carillo et al. for *CALR* mutations, [16]) or product-sizing analysis and Sanger sequencing according to the methods of Klampfl *et al*.[6]. For the technical details of the tests and flowchart, see S1 Flowchart, S1 Table and S1 Text. Methods and reagents.

## Results

A total of 298 ET patients from the haematology laboratory, University Hospital in Dijon, France, were retrospectively studied. Altogether, a *JAK2*V617F or an *MPL* exon 10 mutation was observed in 179 and 13 patients, respectively. Finally 83 ET patients (33 males, 50 females) with a mean age of 63 years at the time of diagnosis (ranging from 20 to 95 years) diagnosed from 2005 to 2013 did not harbour any mutation and were considered “double-negative” and thus tested using three molecular methods for a *CALR* mutation

**Fig 1:**
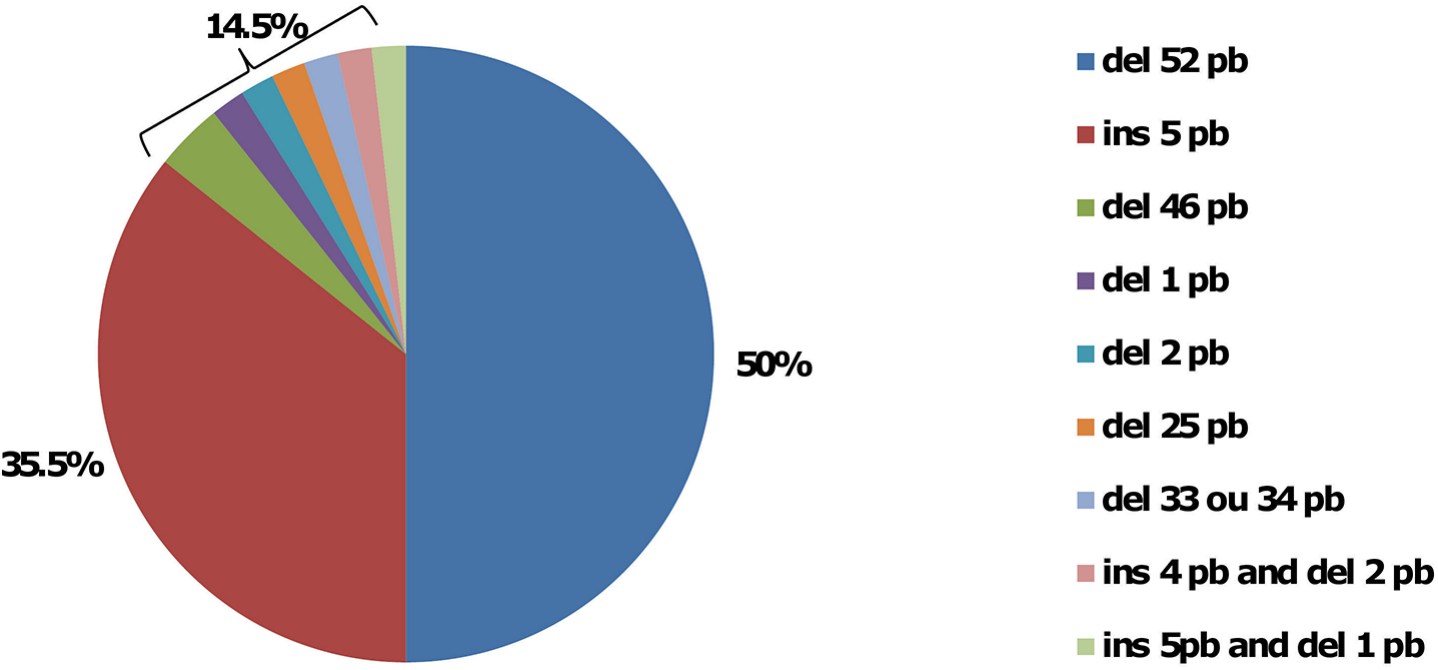
Distribution of different types of *CALR* mutations.

**Fig 2:**
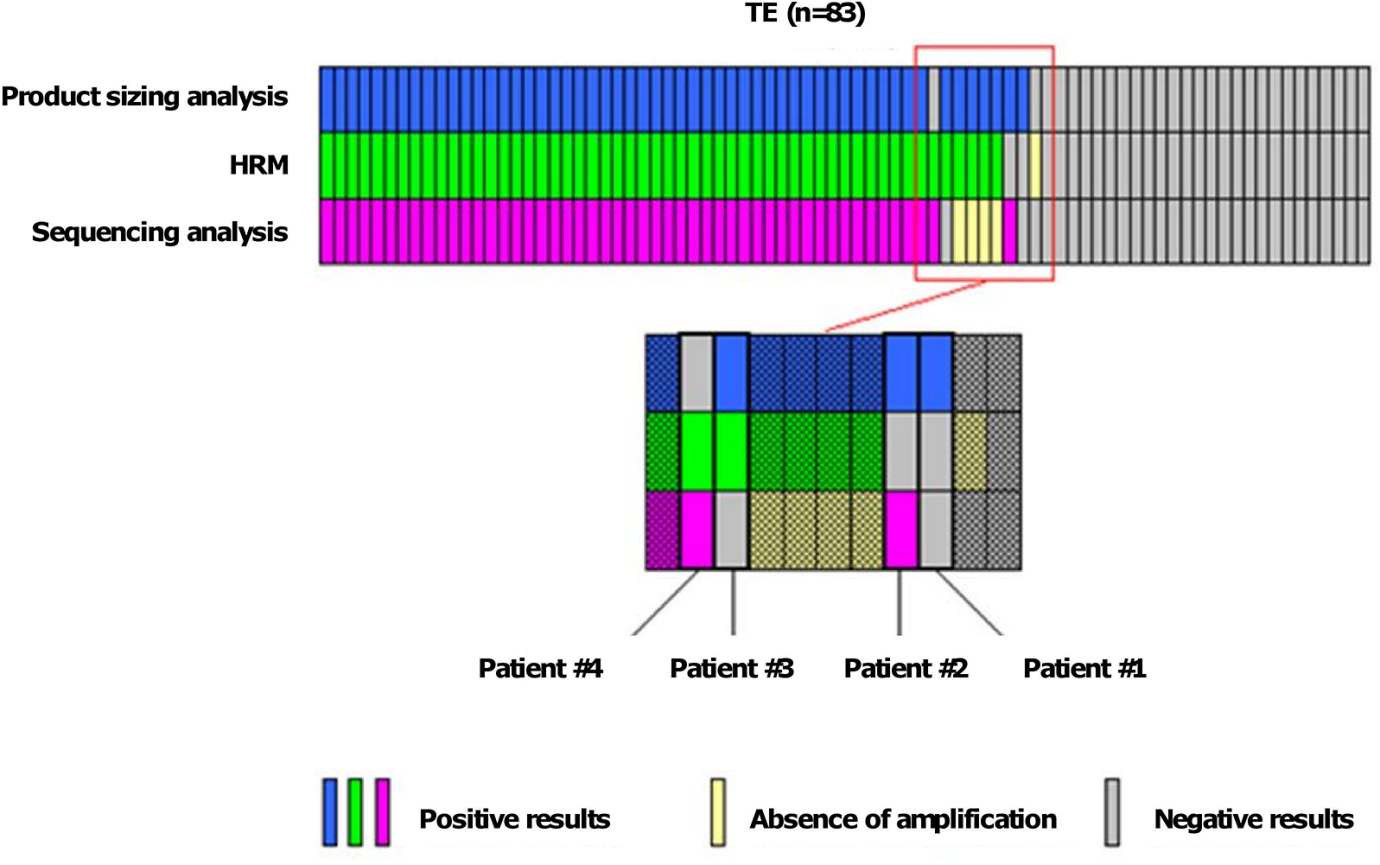
Results of *CALR* mutations among 83 ET patients.

Using the HRM method, a positive curve was noted in 54 patients (65%), whereas no *CALR* mutation was observed in 28 (35%). Of note, in one patient there was no DNA amplification. Using product-sizing analysis, a *CALR* mutation was noted in 55 ET patients, and no mutation was seen in 28. Finally, using Sanger sequencing, 50 ET patients harboured a *CALR* mutation, whereas no DNA amplification was noted in 4 samples. Taken together, a *CALR* mutation was noted in 56 patients and was type 1 (leading to a deletion of 52 bp) or type 2 (leading to an insertion of 5 bp) in 28 (50%) and 20 (35.5%) patients, respectively (Fig 1). The 8 (14.5%) remaining mutations were 2 deletions of 46bp, 1 deletion of 1bp, 1 deletion of 2bp, 1 deletion of 25bp, 1 deletion of 33bp, 2 indel (1 insertion of 4bp and deletion of 2 bp and 1 insertion of 5bp and deletion of 1 bp). A false negative test was observed in 1, 2 and 2 patients in the product-sizing analysis, and HRM and Sanger sequencing methods, respectively (Fig 2 and S1 Table).

**Fig 3:**
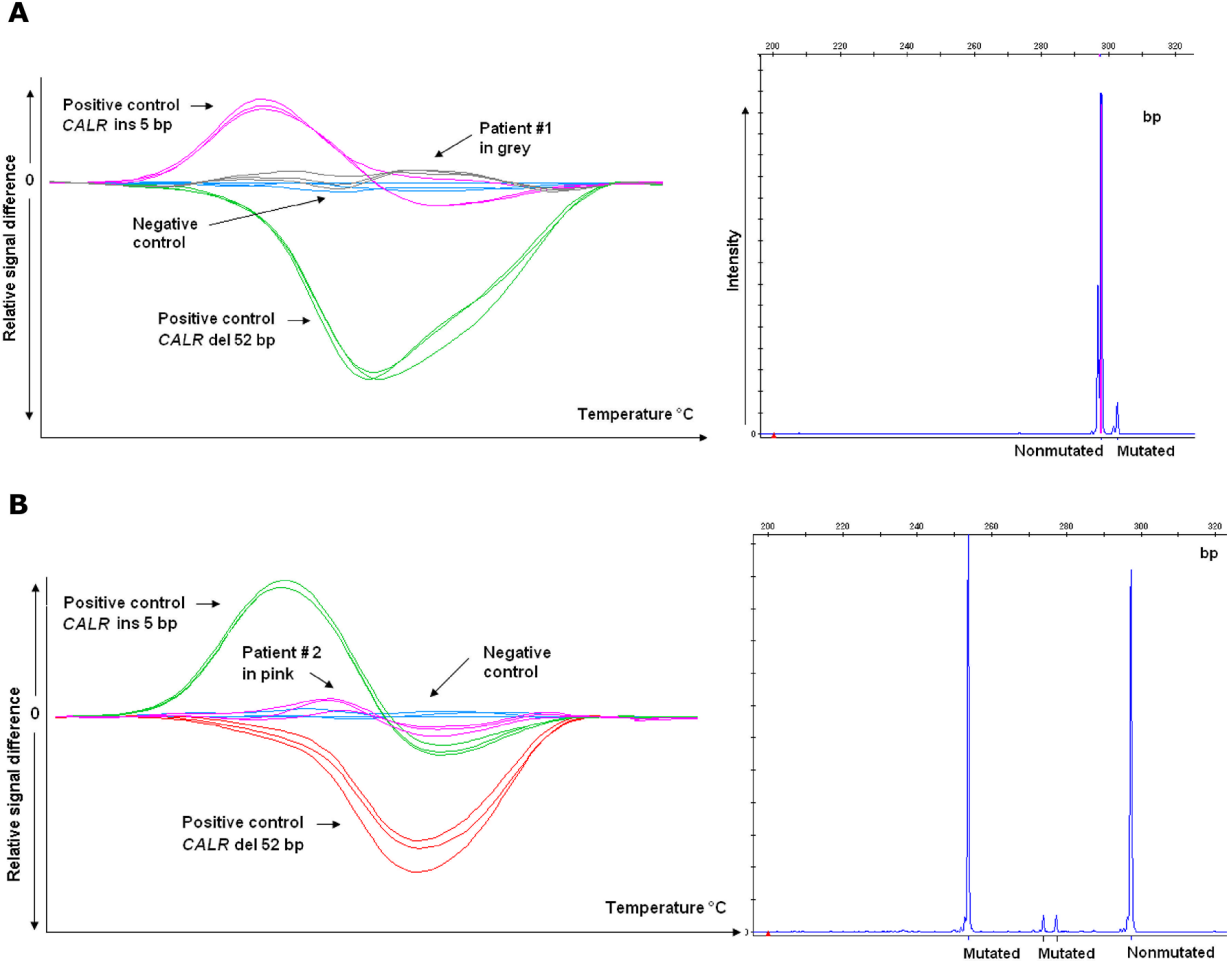
*CALR* mutations determined using High Resolution Melting (HRM) curve analysis and product-sizing analysis from A) patient #1 and B) patient #2.

The two false negative cases in HRM had either negative curves (patient #1) or curves with low amplitude (patient #2) whereas the product-sizing analysis showed the presence of one (patient #1) or several mutant peaks (patient #2) (Fig 3). Of note, in patient #2 the sequencing analysis showed a 25bp deletion associated with an intron mutation.

Both false negative cases in sequencing analysis had positive curves in HRM and mutant peaks in product-sizing analysis, however, with low allelic burden (13% and 8% respectively).

The false negative case in product-sizing analysis had a deletion of 1bp that was not well separated from the wild-type peak in product-sizing analysis despite the fact that HRM curves were positive and the sequencing analysis showed a deletion of 1 bp.

## Discussion

Among 83 ET patients without *JAK2* or *MPL* mutations from a single-centre, 56 had *CALR* mutations (67.5%), a similar frequency to those reported in the literature (from 49 to 71%) [6,7,17]. In the same way, the distribution of the different mutations, i.e. 50% and 35.5% for the 52bp deletion and the 5bp insertion, respectively, was comparable to previously reported rates [6,7,17].

In a wide range of 298 ET patients treated at Dijon University Hospital, the distribution of various mutations (*JAK2*V617F, *MPL* and *CALR*) and "triple-negative" forms (no mutation detected) was 60%, 16,5%, 4,5% and 19%, which is comparable to previous data reported in the literature (64%, 15%, 4% and 16% respectively) [17]. The sensitivity of HRM, Product-sizing analysis and Sanger sequencing was 96.4%, 98.2% and 89.3% respectively, whereas the specificity was 96.3%, 100% and 100% (Table 1). For the HRM test, the cause of the false negative result in two patients was unclear, though it may have been a complex mutation associating insertions and deletions. HRM is usually known for its good sensitivity. Indeed, a recent report noted a 3% allelic burden threshold using an HRM approach for *CALR* mutations [9]. No false negatives were detected in the HRM test, which detected mutations down to 5% [12]. In another study, the HRM test could distinguish between *CALR* type 1 and type 2 mutants with the maximal sensitivity of 2.5% and 1.25%, respectively[10]. In our study, the lower sensitivity of our HRM test could be due to the size of the primer since according to Lim and al. the ideal amplicon length for HRM is usually less than 250 bp [10], whereas in our HRM test the amplicon size was 255 bp compared with 134 bp for the reported study.

**Table 1:** Sensitivity, specificity, time cost and monetary cost of product-sizing analysis, HRM and sequencing analysis. The estimated times do not include DNA extraction, but include the time to prepare the reaction, the reaction time in the analyzer and the time for the analysis with the corresponding software. The monetary cost is estimated for 27 reactions because with one plate of the HRM, 27 reactions can be tested in triplicate.

	Sensitivity (%)	Specificity (%)	Time cost for 27 reactions (day)	Monetary cost for 1 reaction (euros)	Monetary cost for 27 reactions (euros)
Product sizing analysis	98,2	100	1	0,29	7,83
HRM	96,4	96,3	3	0,63	17,01
Sequencing analysis	89,3	100	4	8,64	233,28

As product-sizing analysis can easily distinguish between type 1 and type 2 mutants, the RQ-PCR recently proposed by Chi et al. did not seem necessary [11]. Moreover, the latter technique requires more primers and can only distinguish between type 1 and type 2 mutants. In the same way, Jones et al. [12] (n = 23) recently demonstrated that Next Generation Sequencing (NGS) was the most sensitive method to detect mutations down to mutational burden of 1.25%. However, even though NGS is an accurate method, it is still a costly and time-consuming test compared with HRM and product-sizing analysis. Furthermore, in our experience and according to published data, *CALR* mutations with a low allele burden are rare, which underscores the notion that the use of NGS as a screening test for *CALR* mutations is probably disproportionate.

On the other hand, it has been reported that the allelic burden of *CALR* mutations decreased under treatment, particularly with *JAK2* inhibitors [18] or Interferon [19]. Based on DNA dilutions of a *CALR*-mutated cell line, the limit of detection of *CALR* mutations using product-sizing analysis was evaluated at 5–10% [15]. Since the allele burden of most *CALR* mutations is > 20% and low allele burden *CARL* mutations are rare (in our study, only 1 out of 56 patients had an allelic burden < 13%), we believe that product-sizing analysis providing information on the allelic burden can be useful for monitoring specific therapies.

Finally, from a monetary point of view, product-sizing analysis had the lowest time and monetary costs compared with HRM and Sanger sequencing (respectively 2 and 30 times cheaper, Table 1), thus underscoring its interest as a screening test.

In conclusion, our results suggest that in routine laboratory practice, product-sizing analysis and HRM are overall equally able to detect *CALR* mutations and may be used as first-line screening tests. If these tests are positive, Sanger sequencing can be used to confirm the mutation and determine its type. As HRM is easy and quick, it would be better suited for large series of samples. However, there would be a slightly higher rate of false negative results. Product-sizing analysis provides sensitive and specific results, moreover with a quantitative measurement of *CALR*, which might be useful to monitor specific treatments, and associated with a low cost.

## Supporting Information

S1 Flowchart (TIF)

S1 Table Comparison of the 3 techniques for the detection of CALR mutations in the 83 triple-negative ET patients. NA = no amplification.
(DOCX)

S1 Text Methods and reagents. (DOCX)
