# Supplementary figures and images for: Calreticulin Mutations in Myeloproliferative Neoplasms: Comparison of Three Diagnostic Methods
Source: PLoS One. 2015 Oct 26;10(10):e0141010. doi: 10.1371/journal.pone.0141010 (PMC4621046; doi:10.1371/journal.pone.0141010)

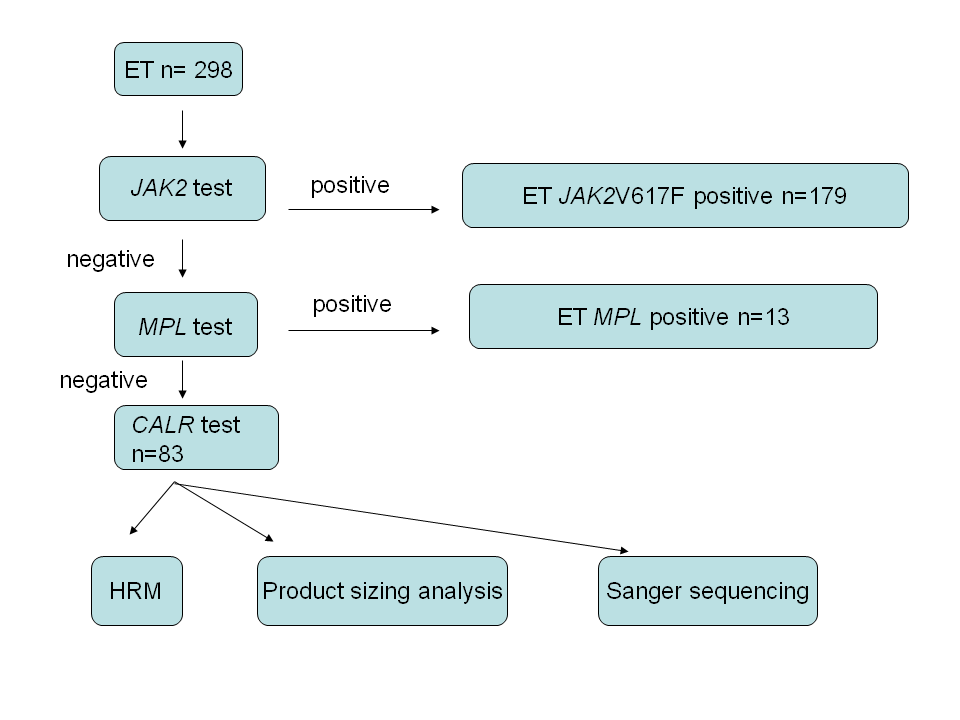

Supplement: S1 Flowchart — (TIF) [file pone.0141010.s001.tif]
