# Supplementary material for: Calreticulin Mutations in Myeloproliferative Neoplasms: Comparison of Three Diagnostic Methods
Source: PLoS One. 2015 Oct 26;10(10):e0141010. doi: 10.1371/journal.pone.0141010 (PMC4621046; doi:10.1371/journal.pone.0141010)
Supplement: S1 Table — NA = no amplification. (DOCX) [file pone.0141010.s002.docx]

| Patient ID | Gender | Age (years) | *CALR* mutations | | | |
| --- | --- | --- | --- | --- | --- | --- |
|  |  |  | HRM | Product-sizing analysis | Sanger sequencing | Allelic burden (Product sizing analysis) in % |
| 1 | F | 55 | Positive | ins 5bp | c1154_1155 ins TTGTC | 37 |
| 2 | F | 65 | Positive | ins 5bp | c1154_1155 ins TTGTC | 51 |
| 3 | F | 45 | Negative | Negative | Negative | 0 |
| 4 | F | 81 | Negative | Negative | Negative | 0 |
| 5 | M | 36 | Positive | ins 5bp | c1154_1155 ins TTGTC | 36 |
| 6 | M | 64 | Positive | del 52bp | Del 52pb/type 1 | 24 |
| 7 | F | 75 | Positive | del 52bp | Del 52pb/type 1 | 50 |
| 8 | F | 32 | Negative | Negative | Negative | 0 |
| 9 | F | 49 | Negative | Negative | Negative | 0 |
| 10 | M | 55 | Positive | del 52bp | Negative | 13 |
| 11 | F | 83 | Negative | Negative | Negative | 0 |
| 12 | F | 66 | Positive | ins 5bp | c1154_1155 ins TTGTC | 43 |
| 13 | M | 73 | Positive | Negative | c1155del or c1156del | 0 |
| 14 | M | 76 | Positive | ins 2 bp | c1150-1151insTGTC c1153_1154del (AA) | 50 |
| 15 | F | 22 | Positive | ins 5bp | c1154_1155 ins TTGTC | 42 |
| 16 | M | 46 | Positive | ins 5bp | NA | 52 |
| 17 | F | 32 | Negative | Negative | Negative | 0 |
| 18 | M | 55 | Negative | Negative | Negative | 0 |
| 19 | F | 77 | Positive | del 52bp | Del 52pb/type 1 | 22 |
| 20 | F | 85 | Negative | Negative | Negative | 0 |
| 21 | F | 53 | Positive | del 52bp | Del 52pb/type 1 | 26 |
| 22 | F | 76 | Positive | ins 5bp | c1154_1155 ins TTGTC | 30 |
| 23 | M | 61 | Negative | Negative | Negative | 0 |
| 24 | F | 47 | Positive | del 52bp | Del 52pb/type 1 | 32 |
| 25 | M | 62 | Positive | del 52bp | Del 52pb/type 1 | 39 |
| 26 | F | 77 | Positive | ins 5bp | c1154_1155 ins TTGTC | 49 |
| 27 | F | 77 | Positive | ins 5bp | c1154_1155 ins TTGTC | 80 |
| 28 | M | 75 | Positive | ins 5bp | c1154_1155 ins TTGTC | 65 |
| 29 | M | 59 | Positive | del 52bp | Del 52pb/type 1 | 53 |
| 30 | F | 67 | NA | del 44,23 and 19 | c1124_1149del | 51 |
| 31 | M | 80 | Positive | del 52bp | Del 52pb/type 1 | 48 |
| 32 | F | 67 | Positive | del 52bp | Del 52pb/type 1 | 55 |
| 33 | F | 50 | Positive | del 52bp | NA | 51 |
| 34 | F | 33 | Negative | Negative | Negative | 0 |
| 35 | F | 20 | Positive | del 52bp | Del 52pb/type 1 | 36 |
| 36 | F | 42 | Negative | Negative | Negative | 0 |
| 37 | M | 57 | Positive | del 52bp | Del 52pb/type 1 | 54 |
| 38 | F | 65 | Positive | ins 5bp | c1154_1155 ins TTGTC | 24 |
| 39 | F | 75 | Negative | Negative | Negative | 0 |
| 40 | M | 62 | Positive | del 33bp | c1102_1135del | 52 |
| 41 | F | 82 | Negative | Negative | Negative | 0 |
| 42 | F | 95 | Positive | del 52bp | Del 52pb/type 1 | 50 |
| 43 | M | 59 | Positive | del 46bp | c1095_1140del | 51 |
| 44 | M | 62 | Negative | Negative | Negative | 0 |
| 45 | F | 86 | Negative | Negative | Negative | 0 |
| 46 | F | 81 | Positive | del 52bp | NA | 43 |
| 47 | M | 80 | Positive | del 52bp | Del 52pb/type 1 | 29 |
| 48 | F | 71 | Positive | ins 5bp | c1154_1155 ins TTGTC | 73 |
| 49 | F | 32 | Negative | Negative | Negative | 0 |
| 50 | F | 68 | Negative | Negative | Negative | 0 |
| 51 | M | 23 | Positive | del 52bp | Del 52pb/type 1 | 50 |
| 52 | F | 30 | Positive | ins 5bp | c1154_1155 ins TTGTC | 16 |
| 53 | M | 61 | Positive | del 52bp | Del 52pb/type 1 | 38 |
| 54 | F | 61 | Positive | del 52bp | Del 52pb/type 1 | 23 |
| 55 | F | 81 | Positive | del 52bp | Del 52pb/type 1 | 60 |
| 56 | M | 58 | Negative | Negative | Negative | 0 |
| 57 | F | 30 | Negative | Negative | Negative | 0 |
| 58 | F | 87 | Positive | ins 5bp | c1154_1155 ins TTGTC | 47 |
| 59 | M | 82 | Negative | Negative | Negative | 0 |
| 60 | M | 60 | Negative | Negative | Negative | 0 |
| 61 | F | 79 | Positive | del 2bp | c1142del c1213del | 50 |
| 62 | F | 41 | Negative | Negative | Negative | 0 |
| 63 | M | 27 | Positive | ins 5bp | c1154_1155 ins TTGTC | 39 |
| 64 | M | 38 | Positive | del 46bp | c1100_1145del | 41 |
| 65 | F | 49 | Negative | Negative | Negative | 0 |
| 66 | M | 63 | Positive | ins 5bp | c1154_1155 ins TTGTC | 51 |
| 67 | F | 71 | Positive | del 52bp | Del 52pb/type 1 | 32 |
| 68 | F | 94 | Positive | ins 5bp | c1154_1155 ins TTGTC | 51 |
| 69 | M | 48 | Positive | ins 5bp | c1154_1155 ins TTGTC | 50 |
| 70 | M | 46 | Positive | del 52bp | Del 52pb/type 1 | 56 |
| 71 | F | 69 | Negative | Negative | Negative | 0 |
| 72 | F | 42 | Negative | ins 5bp | Negative | 8 |
| 73 | M | 63 | Positive | del 52bp | Del 52pb/type 1 | 23 |
| 74 | F | 92 | Positive | ins 5bp | c1154_1155 ins TTGTC | 56 |
| 75 | M | 77 | Positive | ins 5bp | c1154_1155 ins TTGTC | 48 |
| 76 | F | 62 | Positive | del 52bp | Del 52pb/type 1 | 50 |
| 77 | M | 70 | Positive | del 52bp | Del 52pb/type 1 | 65 |
| 78 | F | 67 | Negative | Negative | Negative | 0 |
| 79 | F | 49 | Negative | Negative | Negative | 0 |
| 80 | M | 68 | Negative | Negative | Negative | 0 |
| 81 | M | 80 | Positive | del 52bp | Del 52pb/type 1 | 51 |
| 82 | M | 73 | Positive | del 52bp | NA | 69 |
| 83 | F | 84 | Positive | del 52bp | Del 52pb/type 1 | 25 |

Supplemental Table. Comparison of the 3 techniques for the detection of *CALR* mutations in the 83 triple-negative ET patients. NA = no amplification
