# Supplementary material for: Calreticulin Mutations in Myeloproliferative Neoplasms: Comparison of Three Diagnostic Methods
Source: PLoS One. 2015 Oct 26;10(10):e0141010. doi: 10.1371/journal.pone.0141010 (PMC4621046; doi:10.1371/journal.pone.0141010)
Supplement: S1 Text — (DOCX) [file pone.0141010.s003.docx]

Supplementary file:

Methods and reagents

1. HRM
2. HRM protocol

Screening for mutations was done on 96-well microplates (HRM Light Cycler® 480 Multiwell Plate 96). Each sample was analyzed in triplicate. The reagents used were from the Light Cycler® 480 Resolution Melting Master kit. The following were deposited in each well of the plate, 5 µl of 2X Master Mix, 1.4 µl 25 mM MgCl_2_, 2.1 µl of PCR grade H_2_O, 0.24 µl of forward primer 0.25 µM reverse primer 0.25 µM and 1 µl sample DNA to be tested . The concentration was standardized to 5 to 10ng/µL. Samples with a concentration less than 10 ng/µL were used as such, those whose concentration was higher were diluted with sterile nuclease-free water (Euromedex).

The plate was covered with plastic film Light Cycler® 480 Sealing Foil (Roche) and analyzed in the Light Cycler® 480.

The specific primers used were as follows:

Forward: 5'GGTGTGTGCTCTGCCTGC 3'

Reverse: 5'TCAGGCCTCAGTCCAGCC 3'

It generated an amplicon of 255 base pairs (Eurogentec).

1. PCR program of HRM

The 96-well plate in which the samples and reaction mix were deposited was inserted into the Light Cycler® 480 which provided the course of the PCR program and melting curves.

A first preheating step is performed for 10 minutes.

Then, in the second step, the PCR program started. 60 cycles were carried out, each cycle comprising: 10 seconds at 95 °C to dissociate the DNA strands, 20 seconds at 70 °C for primer annealing, and finally 20 seconds at 72 °C to allow elongation of a complementary DNA strand.

In the third step the melting curves were obtained. This was characterized by:

- 1 minute at 95 °C to separate the double-stranded DNA formed during PCR

- 1 minute at 40 °C to allow the strands to re-pair randomly, thus forming heteroduplexes and homoduplexes if the patient is heterozygous and wild or mutant homoduplex if the patient is homozygous.

- 5 seconds at 65 °C in order to start the step to obtain the melting curves.

- A final step where the temperature is gradually increased (0.02 °C per second) to 95 °C.

- Finally, 1 minute at 40 °C to cool the plate.

1. Sequencing analysis
2. Pre-PCR sequencing

The primers used are as follows:

Forward: 5 CAG ATG AGG CAT CTG ACG AG 3'

Reverse: 5 ATC CAC CCC AAA CC TCC GAA 3'

The following were injected into each PCR tube: 5 µl Buffer 10X Gold, 0.4μl dNTP 2.5 mM, 25 mM MgCl_2_ 4μl, 0.25μl of AmpliTaq Gold DNA polymearse, 0.5μl of forward primer at 0.5 µM, 0.5μl of reverse primer at 0.5 µM, 37.35 µl of H_2_O and 2 µl of 50 ng DNA .

The PCR program comprised various stages:

- A first denaturation step for 5 minutes at 95 °C.

- Then in the second step 35 PCR cycles are carried out: 45 seconds at 95 °C in order to separate the DNA strands, one minute at 65 °C for primer annealing, and finally 2 minutes at 72 °C to allow elongation of a complementary DNA strand.

- And the last step at 72 °C for 10 minutes.

Then a control gel was done. The PCR products migrated on the 2% agarose gel in TBE at 100V for 30 minutes. Then PCR products were quantified with a DNA mass ladder.

It generated an amplicon of 466 base pairs.

1. PCR sequencing

The same primers as those used previously were used.

For each sample, two microtubes were prepared: one with the forward primer and the other with the reverse primer.

The following were injected into each tube: 1 µl Terminator Ready Reaction Mix, 1.5 µl of dilution buffer, 0.5 µl of forward primer or reverse 3.2 µM, 5.5 µl of H_2_O and 1 µl of purified PCR product whose concentration target is 2.5 to 3.5 ng / µl.

The PCR sequencing program was:

- An initial denaturation step for 1 minute at 96 °C.

- Then in the second step 25 PCR cycles were carried out: 20 seconds at 96 °C in order to separate the DNA strands, 30 seconds at 50 °C for primer annealing, and finally 3 minutes at 60 °C to allow elongation of a complementary DNA strand.

- And the last step at 4 °C.

Sequences were purified with the Big Dye Terminator Purification kit. 45μl SAM solution and 10 µl Big Dye Purifier were added to each sample. The mixture was vortexed for 30 min and centrifuged for 2 minutes at 1000 g (RCF).

The products were placed on the sequencer. SeqScape® software v2.6 (Applied Biosystems) was used for the analysis.

1. Product sizing analysis
2. Sample preparation:

The concentration of DNA samples to be tested was standardized at 5 ng/µL. Samples with a concentration of less than 5 ng/µL were used as such, those whose concentration was higher were diluted with sterile nuclease-free water (Euromedex).

1. Reaction mix preparation

The mix is ​​prepared in a sterile tube of 1.5mL.

The following were injected into each microtube: 29.75μL H_2_O, 10 µl of Tp colorless (1X), 3μL MgCl_2_ 1.5 µM, 0.2 µM dNTP 1µl, 0.5μL of forward primer at 0.5 µM, 0.5μL reverse primer of 0.5 µM , 0.25 U of Go Taq (Promega) to 1.25 U and 5 µl of DNA of the test sample whose concentration had been standardized to 5 ng/µL.

The specific primers used are as follows:

Forward: 5 ' GAGGTGTGTGCTCTGCCTG 3'

Reverse: 5 ' AGAGACATTATTTGGCGCGG 3'

It generated an amplicon of 298 base pairs (Eurogentec).

1. PCR Condition

The microtubes containing the samples and the reaction mix were placed in the thermocycler GeneAmp PCR System 9700, which ensures the progress of the PCR program.

The initial denaturation step is carried out for 2 minutes at 95 °C.

Then 35 cycles of amplification on the thermal cycler under specific conditions were performed.

Each cycle was composed as follows:

- A denaturation step of DNA at 95 °C for 45 seconds to obtain the single-stranded DNA form.

- A hybridization step at 60 °C for 1 minute to allow the attachment of the specific primer on the single-stranded DNA template. This primer will allow initiation of elongation by Taq polymerase.

- A DNA elongation step by the taq polymerase at 72 °C for 2 minutes.

The final elongation step was carried out at 72 °C for 5 minutes before cooling and storage at 15 °C.

1. Visualization of PCR products: control gel

PCR products were quantified on agarose gel with a 2% weight marker. For this, 4 µl of Low DNA Mass Ladder, 3 µl of PCR product sample and a buffer solution were mixed and then migrated for about 45 minutes at 90V.

1. Preparation of fragment analysis:

PCR products were diluted so as to have a concentration between 2.5 and 3.5 ng/µl, if necessary.

8.8μl formamide, 0.2μl ROX 500-250 and 1µl PCR product diluted in H_2_O were injected into each microtube.

1. Passage on the ABI Prism 3130 Genetic Analyzer (Applied Biosytem):

The samples thus prepared were passed on the sequencer. The results were analyzed with SeqScape® software v2.6 (Applied Biosystems).
